# Sufficiency of FNAB aspirates of posterior uveal melanoma for cytologic versus GEP classification in 159 patients, and relative prognostic significance of these classifications

**DOI:** 10.1007/s00417-013-2515-0

**Published:** 2013-11-24

**Authors:** Zelia M. Correa, James J. Augsburger

**Affiliations:** Department of Ophthalmology, University of Cincinnati College of Medicine, 260 Stetson Street, Suite 5300, Cincinnati, OH 45267 USA

**Keywords:** Melanoma, Uveal neoplasm, Choroidal melanoma, Cytology, Biopsy, needle/methods, Gene expression profile, Survival prognosis, Melanoma/metastasis

## Abstract

**Objective:**

To determine the relative sufficiency of paired aspirates of posterior uveal melanomas obtained by FNAB for cytopathology and GEP, and their prognostic significance for predicting death from metastasis.

**Methods:**

Prospective non-randomized IRB-approved single-center longitudinal clinical study of 159 patients with posterior uveal melanoma sampled by FNAB in at least two tumor sites between 09/2007 and 12/2010. Cases were analyzed with regard to sufficiency of the obtained aspirates for cytopathologic classification and GEP classification. Statistical strength of associations between variables and GEP class was computed using Chi-square test. Cumulative actuarial survival curves of subgroups of these patients based on their cytopathologic versus GEP-assigned categories were computed by the Kaplan–Meier method. The endpoint for this survival analysis was death from metastatic uveal melanoma.

**Results:**

FNAB aspirates were insufficient for cytopathologic classification in 34 of 159 cases (21.9 %). In contrast, FNAB aspirates were insufficient for GEP classification in only one of 159 cases (0.6 %). This difference is statistically significant (*P* < 0.001). Six of 34 tumors (17.6 %) that yielded an insufficient aspirate for cytopathologic diagnosis were categorized as GEP class 2, while 43 of 125 tumors (34.7 %) that yielded a sufficient aspirate for cytopathologic diagnosis were categorized as GEP class 2. To date, 14 of the 49 patients with a GEP class 2 tumor (28.6 %) but only five of the 109 patients with a GEP class 1 tumor (5.6 %) have developed metastasis. Fifteen of 125 patients (12 %) whose tumors yielded sufficient aspirates for cytopathologic classification but only four of 34 patients (11.8 %) whose tumors yielded insufficient aspirates for cytopathologic classification developed metastasis. The median post-biopsy follow-up time for surviving patients in this series was 32.5 months. Cumulative actuarial 5-year probability of death from metastasis 14.1 % for those with an insufficient aspirate for cytopathologic classification versus 22.4 % for those with a sufficient aspirate for cytopathologic classification (log rank *P* = 0.68). In contrast, the cumulative actuarial 5-year probability of metastatic death was 8.0 % for those with an insufficient/unsatisfactory aspirate for GEP classification or GEP class 1 tumor, versus 45.0 % for those with a GEP class 2 tumor (log rank *P* = 0.005).

**Conclusion:**

This study confirmed that GEP classification of posterior uveal melanoma cells obtained by FNAB is feasible in almost all cases, including most in which FNAB yields an insufficient aspirate for cytodiagnosis. The study also confirmed that GEP classification is substantially better than cytologic classification for predicting subsequent metastasis and metastatic death.

## Introduction

While ophthalmologists commonly regard primary uveal melanoma as a purely ophthalmic disorder, the reality is that this condition is a systemic disease that conveys to the affected person a substantial risk of metastasis and metastatic death. Although multiple clinical [1] and histopathological [2] features of these tumors have been shown to be prognostic of an affected person’s relative risk of developing metastasis, none of them individually or in combination has proven to be particularly robust for identifying which patients in a group will versus will not develop metastasis [1, 3–5]. In recent years, certain chromosomal aberrations (most notably chromosome 3 monosomy) [6, 7] and the gene expression profile (GEP) [8–10] of uveal melanoma cells have been shown to be far superior to clinical and histopathologic prognostic factors for classifying an individual patient’s metastatic risk. Preliminary reports of both chromosomal and transcriptional prognostic factors for metastasis and metastatic death were based largely on tumor specimens obtained post-enucleation. Because most uveal melanomas are managed today by locally destructive tumor therapies and not by enucleation, an alternative method of procuring sufficient and representative specimens of these tumors for chromosomal and/or transcriptional testing must be employed in most cases. Fortunately, fine needle aspiration biopsy (FNAB) has proven to be an effective method for obtaining such tumor specimens, at least from tumors classified by size as medium size or larger [10, 11].

According to published peer-reviewed data, primary uveal melanomas evaluated by GEP cluster into two distinct subgroups [9]. GEP class 1 tumors are low grade melanomas that are associated with low metastatic risk, while GEP class 2 tumors are high grade melanomas that are associated with high metastatic risk. When GEP classification has been studied in head to head comparison with chromosomal testing by various methods, GEP classification proved superior to chromosomal classification for predicting metastatic risk.

This study was conducted with the purpose of determining the relative sufficiency of paired aspirates of posterior uveal melanomas obtained by fine needle aspiration biopsy (FNAB) for cytopathologic classification and gene expression profile (GEP) classification, and to determine the relative prognostic significance of these different classifications for predicting subsequent patient death from metastasis.

## Methods

Our study was a prospective, non-randomized, IRB-approved, single-center longitudinal clinical study of 159 patients with primary posterior uveal melanoma sampled by FNAB between September 2007 and December 2010. All tumors in this series were sampled in at least two separate sites, and most were sampled in four separate sites using previously published FNAB techniques [11, 12]. The first and (when available) third aspirates were suspended into a 50:50 mixture of balanced salt solution and absolute alcohol, and submitted to our pathology laboratory for cytopathologic processing and analysis [13]. The secondary and (when available) fourth aspirates were flushed into a transport vial containing buffered tissue culture medium, snap frozen on dry ice, and submitted to the Harbour laboratory (Department of Ophthalmology and Visual Sciences, Washington University, St. Louis, Missouri) (*n* = 134 cases, 81.3 %) or the Castle Biosciences, Inc., reference laboratory (Phoenix, Arizona) (*n* = 25 cases, 18.7 %) for gene expression profiling and GEP classification. GEP testing on all cases in this series was performed using a previously described PCR-based 15-gene assay comprising 12 discriminating genes and three endogenous control genes [10].

All patient data was retrieved, and all cases were analyzed with regard to sufficiency of the obtained aspirates for cytopathologic classification and GEP classification. Aspirates were classified cytologicaly as insufficient for cytopathologic diagnosis, melanocytic nevus, borderline melanocytic tumor, spindle cell melanoma, mixed cell melanoma, unspecified uveal melanoma, epithelioid melanoma, or necrotic melanoma. Aspirates were categorized by GEP as class 1, class 2, or failed (GEP testing was unable to identify all of the control genes after multiple amplifications).

Cumulative actuarial survival curves of subgroups of these patients based on their cytopathologic versus GEP-assigned categories were computed by the Kaplan–Meier method. The endpoint of this survival analysis was death from metastatic uveal melanoma. Significance of differences between the curves was evaluated using the log rank test. Statistical strength of associations between the studied clinical variables and GEP class of the tumor was evaluated using cross-table analysis with computation of the chi-squared statistic. An alpha level of 0.05 was selected as the cutoff value for assigning statistical significance of differences identified by this analysis. All statistical analyses were performed using Statistical Package for the Social Sciences (SPSS) version 11.1 for Windows.

## Results

Our study group consisted of 159 patients whose ages ranged from 17 to 87 years, with a median age of 60.6 years and mean age of 60.9 years. There was no correlation between patient age and GEP classification of the tumor (*P* = 0.137). The tumors studied had basal diameters that ranged from 4.5 to 22 mm (median 12 mm, mean = 12 mm), and thicknesses from 1.7 to 16 mm (median 5.4 mm, mean = 5.8 mm). Tumors were located anywhere from 0 to 23 mm from the disc (median 5.0 = mm, mean = 5.7 mm) and from 0 to 21 mm from the fovea (median = 4.5, mean = 5.7 mm). The correlation between largest basal diameter and GEP classification of the melanocytic tumors studied is revealed in Table 1. One hundred and fourteen patients (71.7 %) presented tumors that were exclusively choroidal, and 45 patients presented ciliary body involvement. The correlation between tumor involvement (i.e., ciliary body involvement) and GEP classification of the melanocytic tumors studied is revealed in Table 2.

**Table 1:** Largest basal diameter versus GEP classification of 158 patients with a melanocytic lesion sampled by FNAB

Crosstabs	GEP class of tumor	
Largest basal diameter (LBD)	Class 1 [*n* (%)]	Class 2 [*n* (%)]
Small (LDB ≤ 10 mm)	47 (83.9 %)	9 (16.1 %)
Medium (LDB 10 mm < LDB ≤ 15 mm)	47 (63.5 %)	27 (36.5 %)
Large (LBD > 15 mm)	15 (53.6 %)	13 (46.4 %)

*P* = 0.007

**Table 2:** Tumor location versus GEP classification of 158 patients with melanocytic lesions sampled by FNAB

Crosstabs	GEP class of tumor	
Tumor location (ciliary body involvement)	Class 1 [*n* (%)]	Class 2 [*n* (%)]
Exclusively choroidal	87 (77.0 %)	26 (23.0 %)
Involving cilicary body	22 (48.9 %)	23 (51.1 %)

*P* = 0.001

FNAB yielded an insufficient aspirate for cytopathologic classification in 34 of the 159 cases (21.4 %). In contrast, the FNAB aspirates were insufficient (or unsatisfactory) for GEP classification in only one of the 159 cases (0.6 %). This difference is highly statistically significant (*P* < 0.001).In cases of sufficient aspirate for cytological diagnosis, one was classified as a melanocytic nevus (0.6 %), ten were classified as a borderline melanocytic tumor (6.3 %), 37 were spindle cell melanoma (23.3 %), 34 were mixed cell melanoma (21.4 %), eight were unspecified melanomas (5.0 %), and 35 were epithelioid cell or necrotic melanoma (22 %). The tumor in the 158 successful cases was categorized as GEP class 1 in 109 (69.0 % %) and GEP class 2 in 49 (31.0 %) (Table 3).

**Table 3:** Cytologic diagnosis versus GEP class of 159 patients submitted to fine needle aspiration biopsy for cytologic and GEP classification

Cytologic diagnosis	Gene expression profile classification		
	Failed N (%)	Class 1 [*n* (%)]	Class 2 [*n* (%)]
Insufficient aspirate		28 (82.4 %)	6 (17.6 %)
Melanocytic nevus		1 (100 %)	
Borderline melanocytic tumor	1 (10 %)	7 (70 %)	2 (20.0 %)
Spindle cell melanoma		27 (73.0 %)	10 (27.0 %)
Mixed cell melanoma		19 (55.9 %)	15 (44.1 %)
Unspecified melanoma		3 (37.5 %)	5 (62.5 %)
Epitheliod or necrotic melanoma		24 (68.6 %)	11 (31.4 %)
Total	1 (0.6 %)	109 (68.6 %)	49 (30.8 %)

*P* = 0.034

Six of the 34 cases (17.6 %) with an insufficient aspirate for cytopathologic classification proved to be Class 2 tumors by GEP. Four of these six patients have died of choroidal melanoma metastasis to date, and the other two have been seen in recent clinical follow-up. Both patients remain healthy and are metastasis-free. In contrast, 43 of the 125 cases (34.7 %) that yielded a sufficient FNAB aspirate for cytopathologic classification proved to be GEP Class 2 tumors (Table 4).

**Table 4:** Sufficiency of aspirates for cytologic diagnosis versus GEP classification of 158 patients with a melanocytic lesion sampled by FNAB

Crosstabs	GEP class of tumor	
Sufficiency of aspirate for cytology	Class 1 [*n* (%)]	Class 2 [*n* (%)]
Insufficient for diagnosis	28 (82.4 %)	6 (17.6 %)
Sufficient for diagnosis	81 (65.3 %)	43 (34.7 %)

*P* = 0.057

**Fig. 1:**
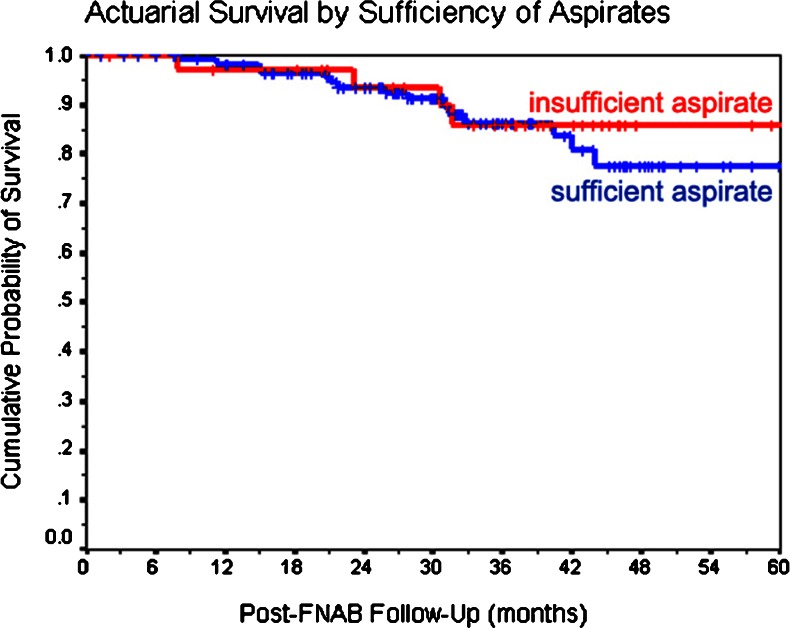
Kaplan–Meier survival curves of 159 patients divided into subgroups according to their sufficiency of aspirate for cytologic diagnosis (insufficient aspirate = 34 patients and sufficient aspirate = 125 patients). (*p* = 0.68)

**Fig. 2:**
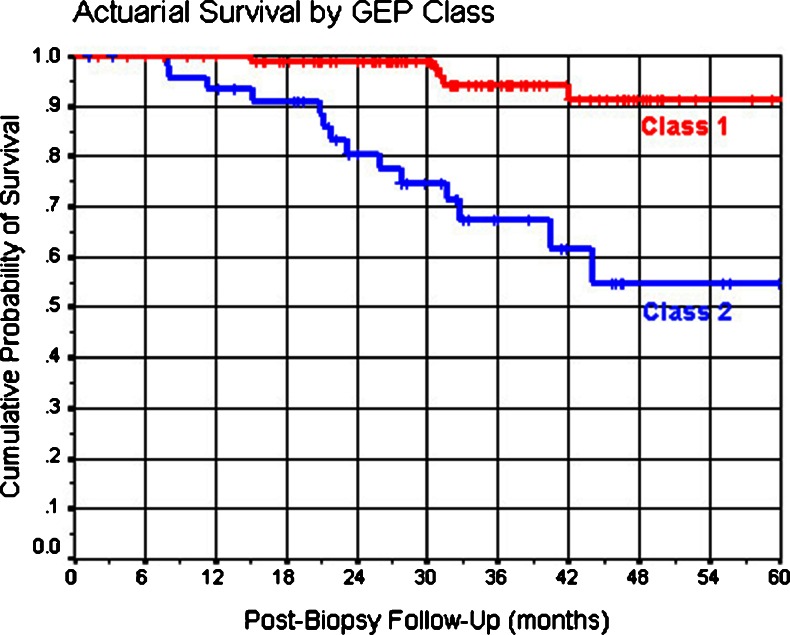
Kaplan–Meier survival curves of 158 patients divided in subgroups according to their GEP classification (GEP class 1 = 109 patients and GEP class 2 = 49 patients). (*p* = 0.005)

Through available follow-up, 14 of the 49 patients (22.4 %) with a GEP class 2 tumor but only five of the 109 patients (1.8 %) with a GEP class 1 tumor have developed metastasis from their uveal melanoma (Table 5). In contrast, 15 of the 125 patients (12 %) with a sufficient aspirate for cytopathologic classification versus four of the 34 cases (11.8 %) with an insufficient aspirate for cytopathologic classification developed metastasis. The median post-FNAB follow-up time among surviving patients in the entire group was 56.4 months (±1.4 months) (95 % CI 53.5–59.2). The median post-FNAB follow-up time of patients with a GEP class 1 tumor was 60.6 months (±1.1 months) (95 % CI 58.3–62.8). Meanwhile, median post-FNAB follow-up time of patients with a GEP class 2 tumor was 45.3 months (±3.1 months) (95 % CI 39.3–51.4). The cumulative 5-year mortality rate from metastasis was 14.1 % for the patients with an insufficient aspirate for cytopathologic classification, versus 22.4 % for the patients with a sufficient aspirate for cytopathologic classification (log rank *P* = 0.68) (Fig. 1). In contrast, the 5-year mortality rate from metastasis was 8.0 % for the patients with insufficient aspirates for GEP or a GEP class 1 tumor, versus 45.0 % for the patients with a GEP class 2 tumor (log rank *P* = 0.005) (Fig. 2).

**Table 5:** Metastatic disease status versus GEP classification of 158 patients with a melanocytic lesion sampled by FNAB

Crosstabs	GEP class of tumor	
Metastatic disease status	Class 1 [*n* (%)]	Class 2 [*n* (%)]
No metastasis detected	104 (95.4 %)	35 (71.4 %)
Metastasis identified	5 (4.6 %)	14 (28.6 %)

*P* < 0.001

## Discussion

Recognizing the substantial potential for primary uveal melanomas to metastasize and kill the affected host (regardless of the method of primary treatment and the apparent local effectiveness of that intervention), ophthalmologists and medical oncologists have long desired a clinically useful method of testing patients with this tumor that (1) has a high probability of classification success in all patients tested, and (2) distinguishes reliably between those at high versus low risk of developing metastasis. Gene expression profiling of the cells of the primary intraocular tumor obtained by FNAB seems to fulfill most of the desires [14, 15].

Because the clinical and cytologic prognostic factors for survival of uveal melanoma patients have been extensively discussed [1, 13, 16, 17], we described these findings in our patient population and attempted to correlate them with GEP. However, our study group is different than most series because it contains substantially more patients with small tumors (largest basal diameter ≤3.5 mm) than other reported studies [18, 19]. This is also reflected by the substantially lower percentage of patients with a Class 2 tumor in our series [8].

Largest basal diameter of the studied tumors presented a significant correlation with GEP classification. Smaller tumors were much more likely to be GEP class 1; however, in the larger tumors the difference in distribution between class 1 and class 2 tumors by GEP was not as distinguishable. Once again, this result may be due to our sample being composed of smaller tumors, and perhaps a larger sample size would show a more significant trend in the distribution GEP class among the medium and larger tumors.

Tumors involving the ciliary body have been known to have a worse survival prognosis [4]. In our series, tumors that were exclusively choroidal were much more likely to be class 1 and consequently carry a better survival prognosis, and there was a trend towards tumors with ciliary body involvement being more likely class 2. This correlation, although statistically significant, probably needs a larger sample size to reveal a more distinctive distribution of tumors involving the ciliary body classified by GEP as class 1 or class 2.

The yield of FNAB is an important topic to be considered. Many authors have pointed out the limited specimen yield of FNAB using smaller gauge needles (25- and 27-G) as a limitation for the widespread use of FNAB. Conversely, FNAB yield for cytology has been reported to be lower among smaller thinner tumors [20]. Although our group has shown that an insufficient aspirate for cytologic diagnosis may be a meaningful result, especially among smaller tumors [21], it is curious to see that GEP testing seems to be feasible in almost all cases, even in very minute specimens, and was able to reveal that a number of cases of insufficient aspirates or diagnosed as nevi proved to be class 2 tumors. However, contrary to our previous experience, the survival of patients whose FNAB yielded an insufficient aspirate for cytoilogy was not significantly different from the group whose FNAB yielded a sufficient aspirate. Reviewing these cases and the cytologic assessment at the time, it was brought to our attention that these studies were performed by a new pathology group, and probably because of their lack of experience with minute specimens, we speculate that the assessment of an insufficient aspirate was made in a substantially larger number of cases.

Furthermore, our study has shown a definite trend in correlation between insufficiency of aspirates and class 1 tumors. An association between absence of epithelioid cells and GEP class 1 tumors cannot be established at this point. Again, definitive correlations may not be present due to our sample size.

This study confirmed that GEP classification of frozen posterior uveal melanoma cells obtained by FNAB is feasible in almost all cases, including most in which FNAB yields an insufficient aspirate for cytopathologic diagnosis. The study also confirmed that GEP classification is substantially better than cytopathologic classification for predicting subsequent metastasis and metastatic death.
